# Ta_2_N_3_ nanocrystals grown in Al_2_O_3_ thin layers

**DOI:** 10.3762/bjnano.8.215

**Published:** 2017-10-16

**Authors:** Krešimir Salamon, Maja Buljan, Iva Šarić, Mladen Petravić, Sigrid Bernstorff

**Affiliations:** 1 Rudjer Bošković Institute, Bijenička cesta 54, HR-10000 Zagreb, Croatia; 2 Department of Physics and Center for Micro- and Nanosciences and Technologies, University of Rijeka, R. Matejčić 2, 51000 Rijeka, Croatia; 3 Elettra-Sincrotrone Trieste, Strada Statale 14, km 163.5, I-34149 Basovizza (TS), Italy

**Keywords:** nanocomposites, multilayers, refractory plasmonics, self-assembly, Ta_2_N_3_ nanoparticles

## Abstract

Tantalum nitride nanoparticles (NPs) and cubic bixbyite-type Ta_2_N_3_ nanocrystals (NCs) were grown in (Ta–N+Al_2_O_3_)/Al_2_O_3_ periodic multilayers (MLs) after thermal treatment. The MLs were prepared by magnetron deposition at room temperature and characterized using grazing incidence small-angle X-ray scattering (GISAXS), X-ray reflectivity (XRR), grazing incidence X-ray diffraction (GIXRD), secondary ion mass spectrometry (SIMS) and X-ray photoelectron spectroscopy (XPS). We found amorphous tantalum nitride NPs at 600–800 °C, with a high degree of ordering along the surface normal and short-range ordering within the layers containing tantalum (metallic layers). At an even higher annealing temperature of 900 °C the NPs crystallize in the rare and relatively unexplored Ta_2_N_3_ phase. However, the environment, morphology and spatial ordering of the NCs depend on the thickness of the metallic layers. For 12 nm thick metallic layers, the Ta_2_N_3_ NCs have an average diameter of 6 nm and they are confined and short-range ordered within the metallic layers. When the metallic layers are thinner, the NCs grow over 20 nm in diameter, show no spatial ordering, while the periodic structure of the ML was completely destroyed. The results presented here demonstrate a self-assembly process of tantalum nitride NPs, the morphological properties of which depend on the preparation conditions. This can be used as a generic procedure to realize highly tunable and designable optical properties of thin films containing transition-metal nitride nanocrystals.

## Introduction

Metallic nanoparticles (NPs) can confine visible light to nano-scale structures via local surface plasmon resonance (LSPR) [1]. LSPR produces a strong near-field enhancement and a local heating [2–3], which are considered to be promising in several applications ranging from surface-enhanced Raman scattering [4], to catalysis [5] and heat-assisted magnetic recording [6]. Currently, research in these fields is dominated by nanoparticles based on noble metals due to their strong plasmonic resonance in the visible part of the electromagnetic spectrum [7]. However, due to the wide range of parameters for separate applications, such as operation at longer wavelengths, high temperature durability, chemical stability, corrosion resistance, low cost or mechanical hardness, there is an increasing interest in various alternative materials that could optimize the device performance for specific plasmonic applications [8–10].

One promising group of alternative plasmonic materials includes the transition-metal nitrides such as TiN, ZrN, TaN or HfN [11–13]. Their advantages are compatibility with the silicon CMOS technology and physical properties suitable for harsh environments (high melting point, chemical stability) [14]. In addition, most of these materials are non-stoichiometric materials (metal or N vacancies) the properties of which can be tuned by using different preparation parameters [15–16]. Along with the choice of material, the LSPR efficiency very much depends on the NP morphology [2,4,17]. For example, TiN nanoparticles can even outperform gold as local heat sources if they are made as nanodisks [3]. Therefore, one of the crucial points in the field of nanoplasmonics is to gain control over the growth and final morphology of NPs.

In this work, we aim to demonstrate a relatively simple preparation process for obtaining isolated nitride NPs within thin dielectric layers. An emphasis is placed here on the control of size and spatial arrangement of NPs, which should then ensure the desired optical properties. This is achieved by using reactive magnetron sputtering and the deposition procedure we already used for the self-assembly of semiconductor quantum dots in amorphous alumina or silica multilayers [18–19]. The key point of this deposition design is the insertion of dielectric layers, so called spacer layers, between all arbitrarily thick active metallic layers. In that way, upon annealing, the growth and final size of nitride NPs could be confined within the metallic layers.

Here we tested the feasibility of this procedure with the tantalum nitride (Ta–N) system, more specifically with the cubic bixbyite type (space group 206, 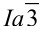) Ta_2_N_3_ nanocrystals embedded within the Al_2_O_3_ matrix. The Ta–N system has a uniquely rich phase diagram with several crystal structures and various stoichiometries [16,20]. The Ta_2_N_3_ phase is a relatively unknown phase, properly described just recently by Ganin et al. [20], and it exhibits some interesting properties [21–22]. In our recent works, we produced mono-layered Ta_2_N_3_ thin films by using the reactive magnetron sputtering deposition technique under conditions that implied a high nitrogen fraction in sputtering gas mixture and post-deposition annealing [23]. We found that the Ta_2_N_3_ phase has metallic properties, which makes it a possible candidate for the LSPR applications. Besides this, the main purpose of this work is to demonstrate a generic preparation route for obtaining well-defined and organized metallic nitride NPs. The results presented here can be used for the production of other promising plasmonic nitrides such as TiN or ZrN. In addition, isolated bixbyite-Ta_2_N_3_ nanocrystals have never been produced so far. Therefore, the specific deposition conditions we describe here could be of interest for a broader research community involved in the relatively complex Ta–N system.

The main effort of this work is to correlate the structure, morphology and composition of tantalum nitride NPs with the processing parameters of the films. For this purpose we used complimentary and well-established characterization techniques such as X-ray reflectivity (XRR), grazing incidence small-angle X-ray scattering (GISAXS), grazing incidence X-ray diffraction (GIXRD), secondary ion mass spectrometry (SIMS) and X-ray photoelectron spectroscopy (XPS).

## Experimental

The films were grown as periodic multilayers by using the magnetron sputter deposition system KJLC CMS-18. In order to obtain tantalum nitride, the depositions were carried out in a reactive atmosphere containing 20% N_2_ and 80% Ar (nitrogen fraction in sputtering gas mixture *p*N_2_ = 0.2) with a total gas pressure of 0.47 Pa. Ta (99.95% purity) and Al_2_O_3_ (99.995% purity) targets were used in dc (15 W) and rf (140 W) operated magnetrons, respectively. The deposition rates were 0.31 nm/s for the Ta target and 0.19 nm/s for the Al_2_O_3_ target. The shutter periodically shielded the Ta target during the 20 s period, which ensured an alternate stacking of “metallic” (Ta–N+Al_2_O_3_) and “spacer” Al_2_O_3_ layers. Nominally, the molar ratio of Ta/Al_2_O_3_ was about 60:40 in the metallic layers. In total, 10 (Ta–N+Al_2_O_3_)/Al_2_O_3_ bilayers were grown on fused silica substrates held at room temperature. After the deposition, the multilayers were annealed for 1 h in vacuum, at several temperatures *T*_a_ ranging from 600 to 900 °C. The spacer layers should suppress the diffusion of Ta atoms between different metallic layers, and thus prevent the formation of too large nitride NPs during the thermal processing. In this work, we show how this assumption is realized in MLs having thicker or thinner metallic layers and for different annealing temperatures. These samples were labelled “ML4mT” and “ML12mT”, where the number indicates the nominal thickness of the metallic layers and “T” indicates the first digit of *T*_a_.

GIXRD measurements were carried out in a diffractometer equipped with a Co X-ray tube while the X-ray patterns were collected with a curved position-sensitive detector placed 120 mm from the sample. A fixed grazing incidence angle of α*_i_* = 1.0° was used for all samples. The multilayer regularity and the thickness of the individual layers were revealed by XRR measurements. XRR was performed on the same set-up used for GIXRD, but with a different detector (Hecus PSD-50M) placed 500 mm from the sample. XRR scans were obtained using a series of diffuse scattering curves as a function of the grazing incidence angle α*_i_* (0° *<* α*_i_* *<* 2.5°, step 0.003°) and then the intensity of the specular reflection, corrected for the background and diffuse component, was determined. The morphological properties of the films were measured with the GISAXS technique. GISAXS patterns were measured at the synchrotron Elettra on the SAXS beamline using a wavelength of λ = 1.54 Å. A 2D detector (Pilatus3 1M) with 16 bit dynamic range was used. A beam stop was placed in front of the detector in the specular plane to protect it from the strong direct beam and strong surface reflection. The sample–detector distance was 1.5 m. For each sample a set of intensity patterns, taken at different grazing incidence angles was recorded.

The chemical bonding of Ta atoms on film surfaces was analyzed by using XPS in a SPECS XPS spectrometer equipped with the Phoibos MCD 100 electron analyzer and a monochromatized source of Al Kα X-rays of 1486.74 eV. The typical pressure in the UHV chamber during analysis was of the order of 10^−7^ Pa. For the electron pass energy of the hemispherical electron energy analyzer of 10 eV used in the present study, the overall energy resolution was around 0.8 eV. All spectra were calibrated by the position of the C 1*s* peak, placed at a binding energy of 284.5 eV. The photoemission spectra were simulated with several sets of mixed Gaussian–Lorentzian functions with Shirley background subtraction [24]. The in-depth distribution of Ta bonds was determined from XPS depth profiles, taken from the bottom of the craters obtained by 2 keV Ar^+^ beam bombardment at normal incidence, rastered over an area of 10 × 10 mm^2^. The compositional in-depth profiles were obtained by SIMS in a Hiden SIMS Workstation, using either 3 keV O_2_^+^ or 5 keV Cs^+^ sputtering ion beams at an impact angle of 45°, and collecting either Al^+^ and Ta^+^ or N^−^, O^−^ and TaN^−^ secondary ions as a function of the sputtering time. The time scale was converted to the sputter depth scale by using the corresponding thickness of the MLs as determined by XRR analysis.

## Results and Discussion

### GISAXS and XRR

**Figure 1:**
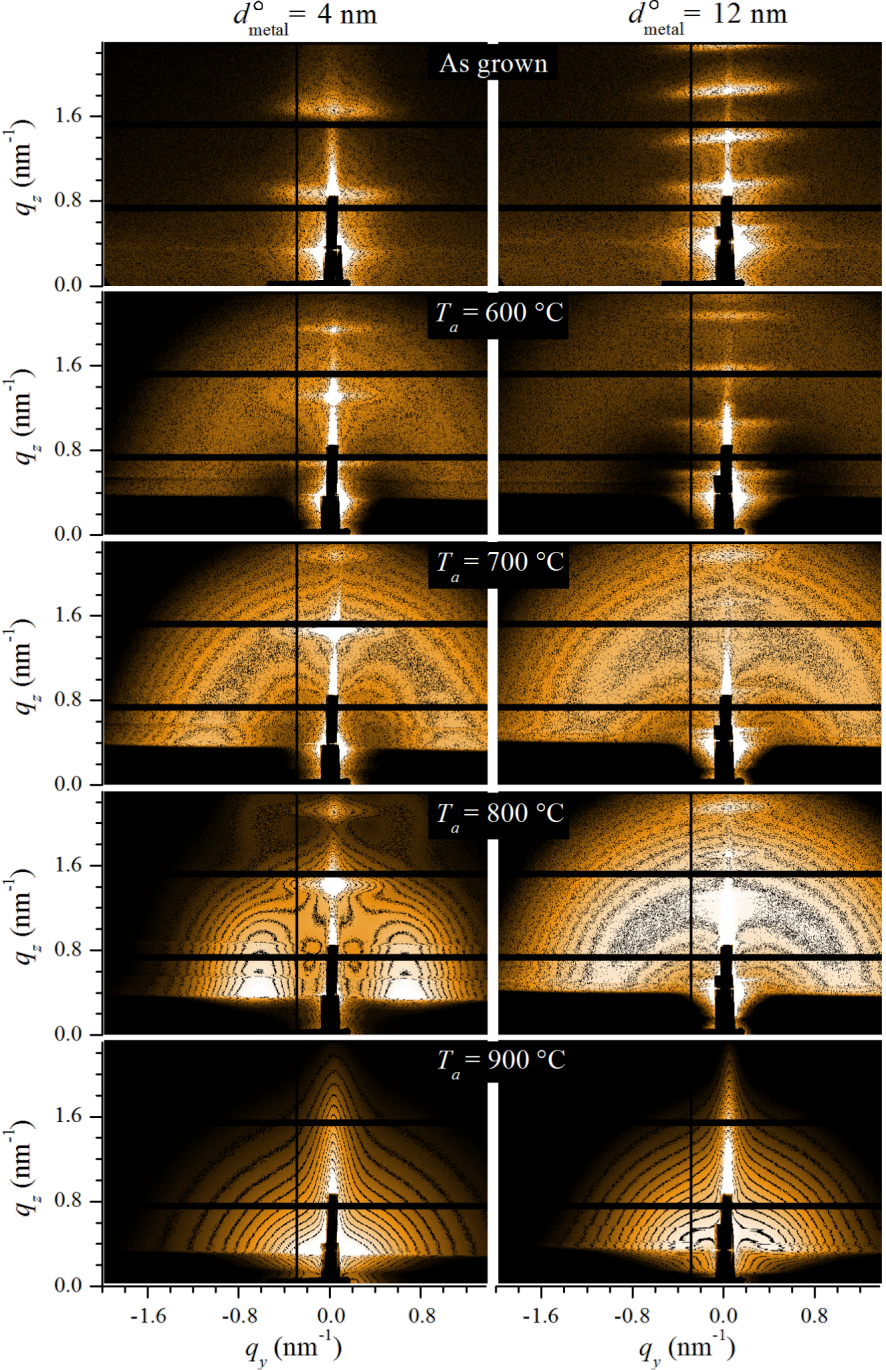
GISAXS patterns of as-grown and annealed MLs with the thinner (left column) and thicker (right column) metallic layers. The vertical and horizontal black lines are non-counting gaps between the segments of the 2D detector.

We investigate the formation, size and arrangement properties of tantalum nitride nanoparticles within an Al_2_O_3_ matrix. GISAXS is especially suitable for this kind of analysis, because it allows for the precise determination of the morphology and spatial correlations of the nanoparticles with excellent statistics [18]. Figure 1 shows the GISAXS patterns of two types of examined multilayers. The ML4m and ML12m multilayers are shown in the left and right columns, respectively, with the annealing temperature increasing down the rows. The patterns of as-grown films (first row) exhibit a range of parallel Bragg sheets centred in the specular plane (*q_y_* = 0). These kind of scattering maxima are usually observed from periodic multilayers with correlated roughness among the interfaces [25–26]. The angular positions of the Bragg sheets yield the periodicity of the corresponding multilayers: 8.5 nm and 16.1 nm for ML4m and ML12m, respectively. Importantly, small angle scattering due to volume density fluctuations was not observed for these films, demonstrating density homogeneity in the as-grown metallic and spacer layers.

Upon annealing at 600 °C and higher, as shown in rows 2–5 of Figure 1, the GISAXS patterns reveal various types of additional scattering such as rings, lobes or strong scattering concentrated in very small angles for the MLs annealed at 900 °C. These small-angle scattering patterns clearly demonstrate a thermally induced segregation of the Ta(N) and Al_2_O_3_ components in the metallic layers, that is, the nucleation and growth of Ta-based NPs. At 600 °C, the GISAXS signal is weak due to a low density contrast between the initially formed Ta clusters and the surrounding media, which is presumably still rich in tantalum. The GISAXS signal increases in intensity and shrinks towards smaller angles with increasing annealing temperature. This indicates an enlargement of the NPs and consequently a stronger segregation of the Ta and Al_2_O_3_ components in the metallic layers resulting in a higher density contrast. Note that up to 800 °C the original Bragg sheets persist and consequently the periodic ML structure was maintained. This means that the NP formation is confined within the metallic layers, at least up to 800 °C. At 900 °C, the Bragg sheets disappeared for both types of multilayers, indicating a loss of the initial regularity along the surface normal or a loss of topography conformation among adjacent interfaces. In any case, it is important to note that annealing promotes the formation of NPs, the morphology of which depend on the temperature as well as on the thickness of the metallic layer.

A more detailed information about the shape and size of the NPs was retrieved from the numerical analysis of the measured GISAXS patterns. From each GISAXS pattern 1D scattering profiles were extracted along *q_y_* (at constant *q_z_* = 0.46 nm^−1^) and along *q_z_* (at constant *q_y_* = 0.29 nm^−1^). These profiles were then independently analysed within a model that assumes a sphere form factor with log–normal size distribution and Hosemann’s paracrystal structure factor for the description of the NP positional ordering [27]. Representative 1D scattering profiles with the corresponding fitting curves are available in Supporting Information File 1 (Figure S1). The results of the numerical analysis are shown in Table 1, where the average dimensions (separations) of the NPs in the two characteristic directions are denoted as *D_y_* (*L_y_*) and *D_z_* (*L_z_*).

**Table 1:** Results of the numerical analysis of GISAXS patterns and XRR curves for the MLs prepared with two different nominal thicknesses for the metallic layers 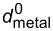 and annealed at different temperatures *T*_a_. The average dimensions (separations) of nanoparticles in the directions parallel and perpendicular to the surface of the film are denoted as *D_y_* (*L_y_*) and *D_z_* (*L_z_*), respectively, and were derived from the GISAXS patterns. The thickness of metallic layers *d*_metal_ and the thickness of the spacer layers 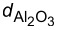 were obtained from XRR data.

sample	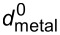 (nm)	*T*_a_ (°C)	*D_y_* (nm)	*D_z_* (nm)	*L_y_* (nm)	*L_z_* (nm)	*d*_metal_ (nm)	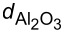 (nm)

ML4m	4	—	—	—	—	—	4.6 ± 0.2	3.7 ± 0.2
ML4m6	4	600	2.2 ± 0.2	1.9 ± 0.3	3.7 ± 0.2	3.1 ± 0.3	4.8 ± 0.3	3.2 ± 0.3
ML4m7	4	700	3.0 ± 0.2	2.3 ± 0.3	4.2 ± 0.2	3.1 ± 0.3	4.7 ± 0.3	3.4 ± 0.3
ML4m8	4	800	4.5 ± 0.2	4.2 ± 0.3	7.3 ± 0.2	8.7 ± 0.3	5.4 ± 0.6	3.2 ± 0.6
ML4m9	4	900	9.2 ± 0.6	8.5 ± 0.8	—	—	—	—
ML12m	12	—	—	—	—	—	12.7 ± 0.3	3.2 ± 0.3
ML12m6	12	600	1.8 ± 0.2	1.6 ± 0.3	3.1 ± 0.2	2.4 ± 0.3	12.8 ± 0.3	3.1 ± 0.3
ML12m7	12	700	2.3 ± 0.2	1.8 ± 0.3	3.3 ± 0.2	2.8 ± 0.3	12.5 ± 0.3	3.3 ± 0.3
ML12m8	12	800	3.5 ± 0.3	3.0 ± 0.3	4.1 ± 0.3	3.9 ± 0.3	12.9 ± 2.0	2.9 ± 1.0
ML12m9	12	900	5.8 ± 0.3	4.9 ± 0.3	12.1 ± 0.3	10.2 ± 0.3	14.4 ± 2.0	2.4 ± 1.1

As we already concluded after visual inspection of the GISAXS patterns, the volume of the NPs increases with *T*_a_. Another important feature of the NPs is their anisotropy. The in-plane dimension (parallel to the surface plane), *D_y_*, is larger than the out-of-plane dimension (perpendicular to the surface plane), *D_z_*, that is, the NPs are slightly elongated in the surface plane. Note that up to 700 °C the morphological properties of the ML4mT and ML12mT samples are similar and with sizes smaller than the thickness of metallic layers. This explains the isotropic scattering rings in the corresponding GISAXS patterns due to 3D short-range inter-particle correlations within the individual metallic layers. However, for *T*_a_ ≥ 800 °C, the size and short-range correlation of the NPs depend on the thickness of the metallic layers. At *T*_a_ = 800 °C, thinner metallic layers comprise larger and more separated NPs. Moreover, the side lobes in the corresponding GISAXS pattern reveal only an in-plane short-range correlation, because the out-of-plane dimension of the NPs, *D_z_*, is comparable to the thickness of the metallic layer *d*_metal_. In other words, each metallic layer comprises only a 2D array of NPs. In the ML12m8 sample, on the other hand, the ratio *d*_metal_/*D_z_* is about four, and therefore short-range ordering is still possible in all directions. This is evident through the appearance of a scattering ring in the corresponding GISAXS pattern. Finally, at *T*_a_ = 900 °C, in the ML4m9 sample the growth of NPs is much faster, reaching an average size that is considerably larger than the thickness of the metallic layers. This can be understood through the coalescence of NPs that were grown in adjacent metallic layers and, consequently, the regular ML structure is destroyed. On the contrary, thicker metallic layers at 900 °C contain smaller NPs and the *d*_metal_/*D_z_* ratio is about two. This indicates that the NPs are still confined within the individual metallic layers in the ML12m9 sample. However, a fully reliable conclusion about the regularity of NP arrangement for this ML could not be drawn from our GISAXS pattern alone, since, as we noted above, the Bragg sheets are absent.

**Figure 2:**
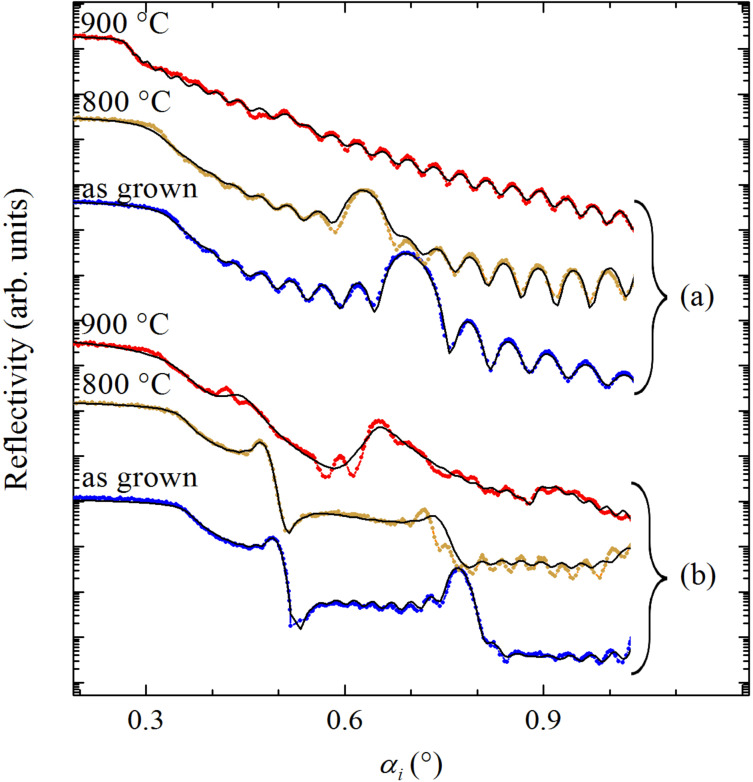
XRR curves of as-grown and annealed (Ta–N+Al_2_O_3_)/Al_2_O_3_ multilayers for two different nominal thicknesses of the metallic layers: (a) 4 nm and (b) 12 nm. The curves are vertically shifted for clarity.

The regularity of the multilayers, or more precisely the density variation along the surface normal, was determined by XRR. Figure 2 shows the XRR spectra for the as-grown and annealed MLs with thinner (upper set, Figure 2a) and thicker (lower set, Figure 2b) metallic layers. The solid black lines are fitting curves that were obtained by the standard Parratt algorithm [28] and roughness according to the Nevot and Croce model [29]. In the simulations we assumed a model with the nominal multilayer structure, while the periodicity, thickness of the metallic and spacer layers, their densities and roughness were varied. The last two columns of Table 1 show the results for two important structural parameters: the thickness of the metallic and the spacer layers. For the as-grown films a good match for the model curves was obtained, with the structural parameters in good agreement with the nominal ones. Moreover, the interface roughnesses are relatively small in these films, showing that the depositions resulted in minimal imperfections of the layers. For the films annealed up to 800 °C we observed a slight increase in interface roughness and a slight decrease in density contrast between the adjacent layers for both types of MLs. Furthermore, clear Bragg peaks, although less intense than in the as-grown MLs, indicate a periodic structure in these samples, in agreement with the GISAXS results. However, at 900 °C, the structural changes are much more prominent, especially in the ML4m9 sample (Figure 2a). There, the Bragg peak is barely visible, indicating intermixing of metallic and spacer layers and consequently a homogenization of the ML stack. On the contrary, for the ML with thicker metallic layers at 900 °C, the Bragg peaks are still visible, although with reduced intensity. We note that for the ML12m9 sample, the fitting curve (see Figure 2b) does not fully match the experimental data. Various alternative parameter sets lead to a similar quality of the fit. Therefore, the corresponding parameters presented in Table 1 have relatively large errors. The reason for that might be slightly different thicknesses of the spacers and metallic layers along the ML stack due to a high diffusion rate at *T*_a_ = 900 °C. The inclusion of fluctuations in the layer thicknesses in the model would inevitably complicate the analysis. However, it would be also without influence on the main result: The periodic structure is more or less preserved in the ML12m9 sample. At the same time, due to the high diffusion rate and nanoparticle growth, the interface topography changes as well. These stochastic rearrangements of interfaces are completely independent from layer to layer, resulting in the loss of vertical correlations among the interface topographies. This explains the absence of Bragg sheets in the GISAXS pattern for the Ml12m9 sample in Figure 1. In any case, according to the XRR and GISAXS results, the nitride nanoparticles in the ML12m9 sample are more or less confined within the separated metallic layers.

### GIXRD

**Figure 3:**
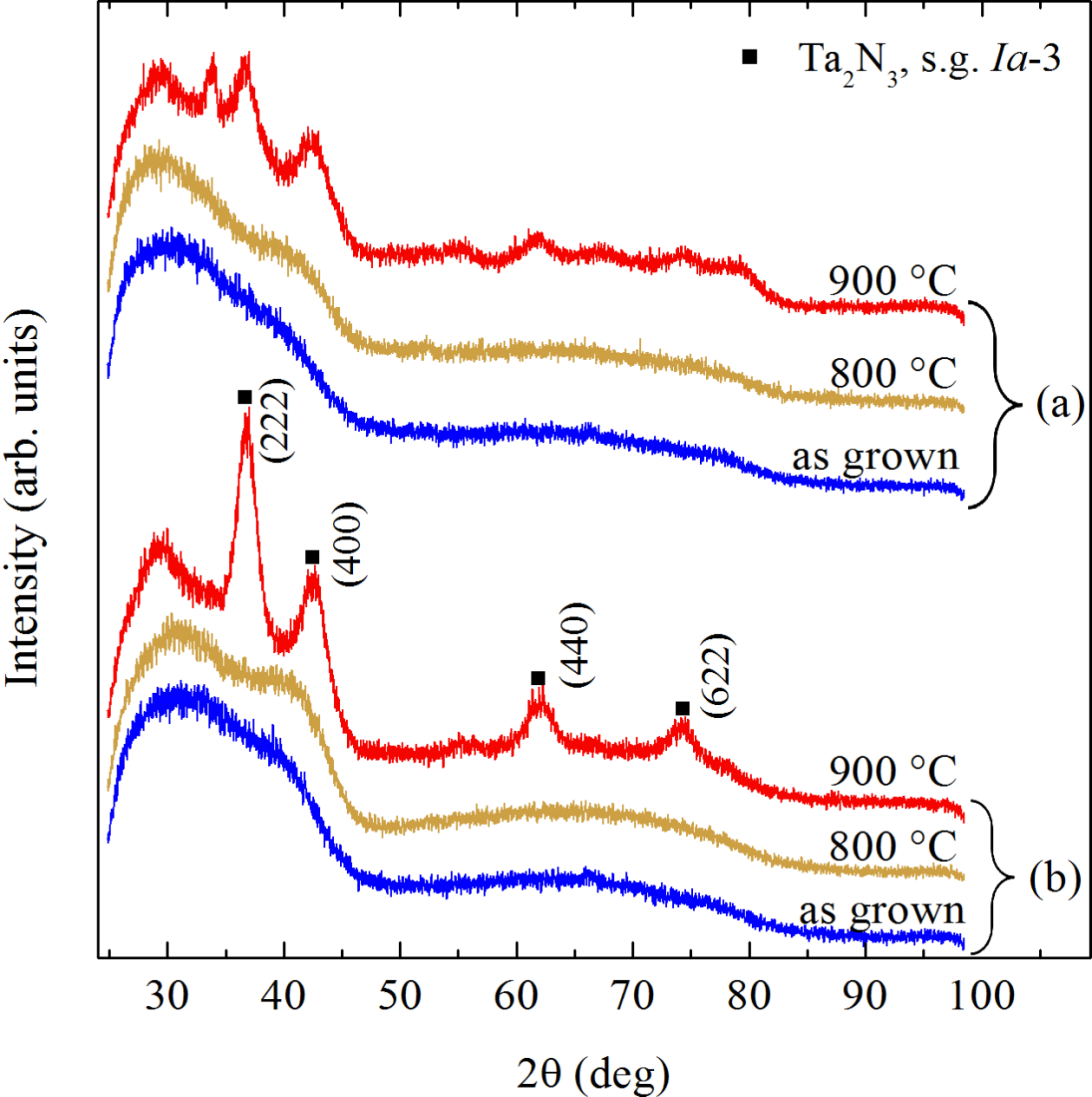
GIXRD curves of as-grown and annealed (Ta–N+Al_2_O_3_)/Al_2_O_3_ multilayers with two different nominal thicknesses of the metallic layers: (a) 4 nm and (b) 12 nm. The curves are vertically shifted for clarity.

The GIXRD study shows that the as-grown MLs and all MLs annealed up to 800 °C were amorphous (Figure 3). Only annealing at 900 °C leads to the crystallization of MLs. The diffraction peaks of the ML12m9 sample match those of the bixbyite-Ta_2_N_3_ phase with a lattice constant of 9.83 Å, while the ML with thinner metallic layers comprises a mixture of Ta_2_N_3_ and Ta_2_O_5_ phases. Taking into account the relatively low *p*N_2_ (0.2) used in the present study and our recent results showing the Ta_2_N_3_ phase only in films deposited under high nitrogen fraction in sputtering gas mixture [16], the appearance of this phase is surprising. Still, it could be understood through the influence of the Al_2_O_3_ component on the coordination and mobility of the tantalum atoms. Indeed, the tantalum nearest-neighbour distance in the as-grown films is relatively large, even 20% larger than that found in the amorphous Ta–N films obtained under high nitrogen fraction in sputtering gas mixture [16]. Such coordination of tantalum atoms presumably leads to the formation of a tantalum nitride phase with a lower density.

It is important to note that the diffraction patterns for both MLs annealed at 900 °C are in fact the superposition of diffraction peaks and a broad maximum. This indicates that only a part of the tantalum atoms is forming Ta_2_N_3_ NCs while the rest still remains in the amorphous configuration.

### SIMS

**Figure 4:**
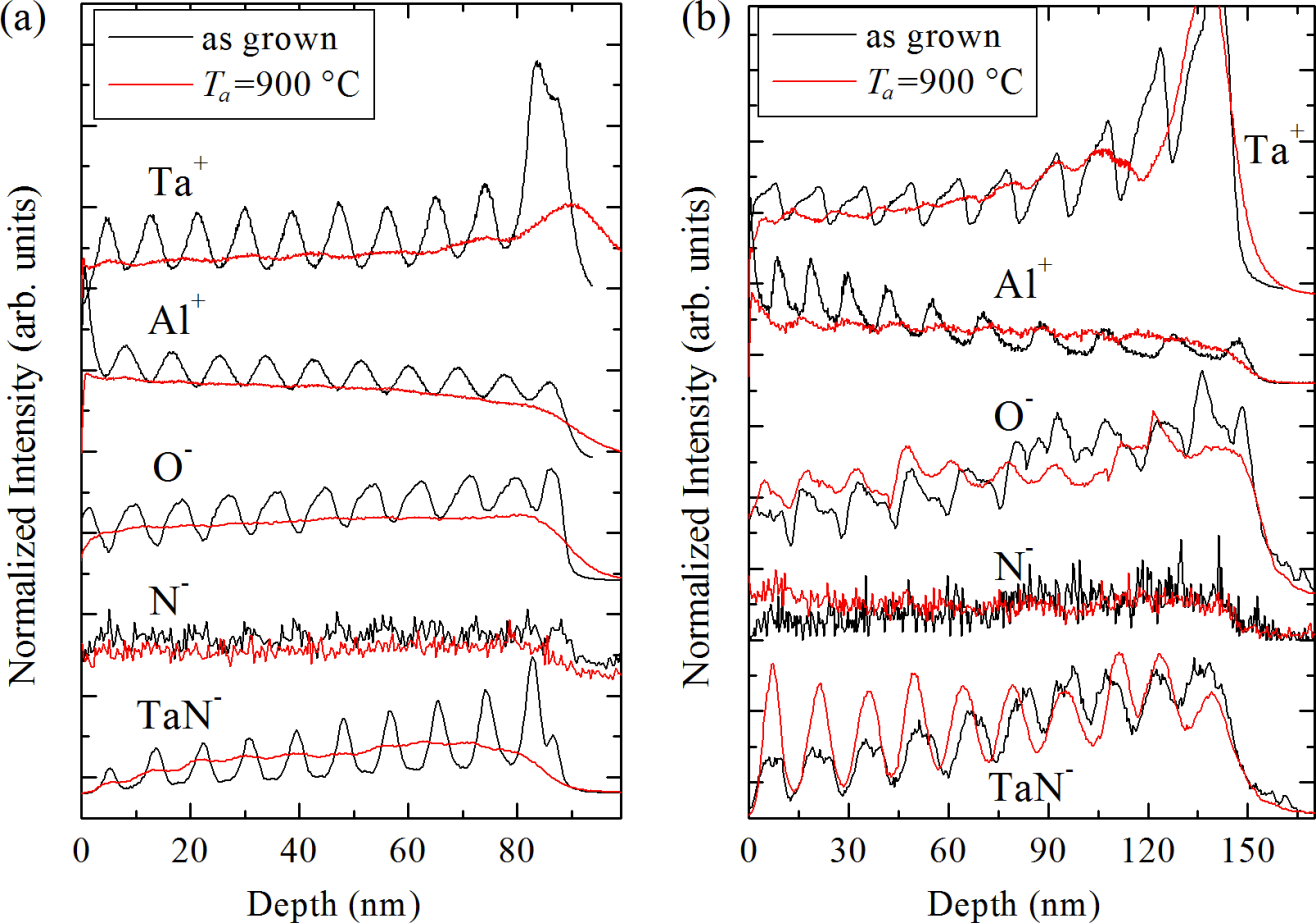
SIMS profiles of as-grown and annealed MLs for two different nominal thicknesses of the metallic layers: (a) 4 nm for ML4m and ML4m9 and (b) 12 nm for ML12m and ML12m9.

SIMS in-depth profiles of the as-grown MLs and MLs annealed at 900 °C are shown in Figure 4. Figure 4a and Figure 4b show profiles for MLs having thinner (ML4m and ML4m9) and thicker (ML12m and ML12m9) metallic layers, respectively. In order to compare the profiles of different ion signals, each profile has been normalized to its average value and then arbitrarily shifted vertically for clarity. The alumina and metallic layers in the as-grown MLs are clearly resolved by the periodic modulation of the Ta^+^, Al^+^ and O^−^ signals. The signals of N^−^ ions are relatively weak and noisy, but one can still notice that the N^−^ profile follows the modulations of the Ta^+^ profile for the as-grown ML with thinner metallic layers (Figure 4a). Most importantly, the intensive TaN^−^ profile is in phase with the Ta^+^ profile, providing a strong evidence for the incorporation of nitrogen in the metallic layers of the as-grown MLs.

All films annealed up to *T*_a_ = 800 °C exhibit oscillating SIMS profiles (not shown) and, therefore, a periodic structure, which is in full agreement with the XRR and GISAXS results from the same films. However, in the MLs annealed at 900 °C (Figure 4), we observed significant changes in the mass distribution within the films. In the ML having thin metallic layers, the periodic distribution of Al, O and N atoms seems to be lost, since the SIMS signals of Ta^+^ and TaN^−^ show only weak variations in intensity, with no regularities or phase coherence with the corresponding signals of the as-grown film (see Figure 4a). On the other hand, in the ML with thicker metallic layers, shown in Figure 4b, the elemental distributions still exhibit periodic structures. However, the confinement of Ta and Al_2_O_3_ to the initially deposited metallic and spacer layers are much less pronounced. In other words, some of the Ta atoms diffuse to the alumina spacer layers, while a part of the alumina diffuses to the metallic layers. Importantly, the TaN^−^ profile of the ML12m9 sample (Figure 4b) shows well-developed oscillations with an amplitude even more pronounced than in the ML12m sample. Therefore, the SIMS measurements from Figure 4b confirm that the Ta_2_N_3_ nanocrystals, which we found with the X-ray experiments, were grown and ordered within the metallic layers. This is not the case for the thinner metallic layers from Figure 4a, which show no similar ordering along the surface normal. The SIMS results are in full agreement with the XRR results for both MLs at 900 °C, which show a periodic modulation of electron density along the surface normal for the thicker ML but not for the thinner ML.

### XPS

**Figure 5:**
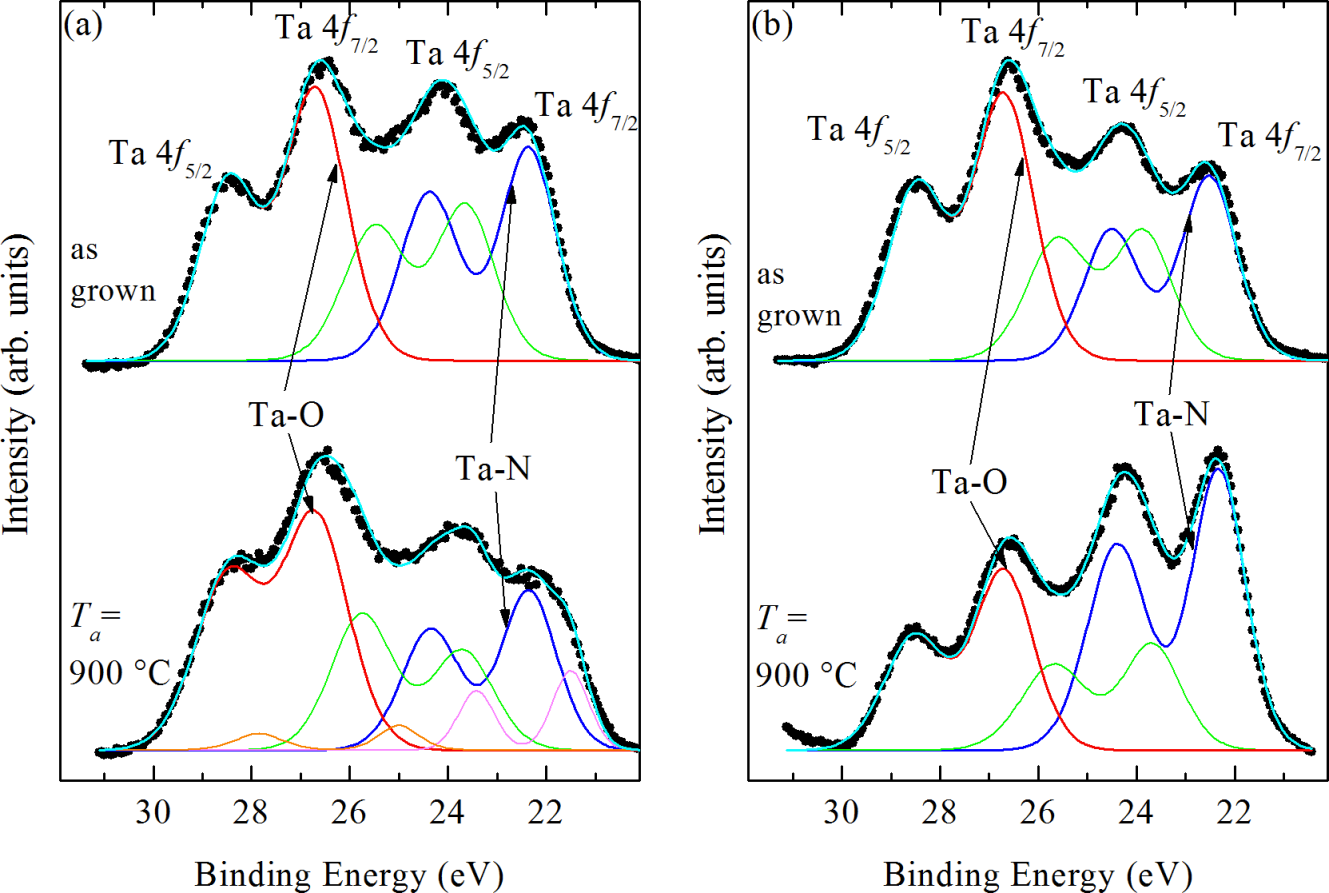
Deconvoluted XPS spectra for Ta 4f core level of as-grown (upper curves) and annealed at 900 °C (lower curves) MLs for two different nominal thicknesses of the metallic layers: (a) 4 nm (b) 12 nm.

To further characterize the chemical states of tantalum within the MLs, we analyzed all samples with XPS in-depth profiling at several depths. The information about Ta bonding to N atoms was obtained from the chemical shifts in the photoemission spectra around the Ta 4f, Ta 4p or N 1s core levels. We focus our XPS measurements on the Ta 4f photoemission, which shows a more distinctive structure and larger chemical shifts than the emissions from the Ta 4p and N 1s levels [30–31]. As an example, we show in Figure 5 the Ta 4f core level photoemission spectra taken from the as-grown and annealed MLs with 4 nm (Figure 5a) and 12 nm (Figure 5b) nominal thickness of the metallic layers. These spectra were collected from the bottom of a crater obtained with Ar^+^ sputtering for 45 min, i.e., approximately at the depth of the first metallic layer (ca. 6 nm). The Ta 4f emission is normally characterized by two peaks characteristic for the spin–orbit splitting of the Ta 4f energy level into the 4f*_7/2_* and 4f*_5/2_* levels, respectively. Therefore, we use two sets of spin–orbit doublets to fit our XPS results. The first doublet at binding energies, BE, of 22.5 and 24.3 eV corresponds to the emission from Ta–N bonds, while the second doublet at BE of 26.6 and 28.5 eV corresponds to a higher oxidation state of tantalum, we assign it to the Ta–O bonds [32]. Furthermore, the Ta 4p and the N 1s photoemission (Figure S2 in Supporting Information File 1) imply the bonding of nitrogen to tantalum, in full agreement with the Ta 4f photoemission. The existence of the Ta–N bonds in XPS is consistent with the SIMS results showing a TaN signal within the metallic layers. On the other hand, the Ta–O bonds are expected in metallic films in the presence of oxygen, as metals prefer to form oxide bonds due to the low heat of oxide formation. As the metallic layers of the as-grown MLs are a homogeneous mixture of Ta, N and Al_2_O_3_, Ta–O bonds are expected. Most importantly, the metastable Ta–N bonds still form and remain stable in the presence of oxygen.

A good fit of the XPS spectra of the as-grown MLs in Figure 5 requires an additional doublet between the Ta–N and Ta–O doublets, which we attribute to the formation of oxynitrides and/or suboxides [32]. The relative amount of different Ta bonds can be determined from fitting the XPS emission curves in Figure 5. The fraction of each chemical state of Ta is then determined from the area of corresponding fitting curves, normalized to the total area of the Ta 4f peak. In that way, we found that the fraction of Ta–N bonds in ML4m and ML12m is 33% and 30%, respectively, while the corresponding fractions for the Ta–O bonds are 41% and 44%, respectively.

The XPS profiles of annealed MLs show a quite similar structure as the as-grown MLs up to *T*_a_ = 800 °C. However, at *T*_a_ = 900 °C, the relative intensities of the Ta–N and Ta–O doublets change noticeably, but with a different ratio of Ta–N to Ta–O bonds in the two types of MLs. The ML with thicker metallic layers (Figure 5b) exhibits the higher intensity for the Ta–N doublet, indicating a more prominent formation of Ta–N bonds. On the contrary, the ML with thinner metallic layers (Figure 5a) exhibits a dominant contribution of Ta–O bonds. Consistently, the relative intensity of the N 1s peak increases for the ML12m9 sample and decreases for the ML4m9 sample (Figure S2 in Supporting Information File 1). These XPS results for annealed samples are consistent with the SIMS profiles, indicating an enhancement of nitride formation in thicker metallic layers and a mixing of alumina and metallic layers in ML with thinner metallic layers (see Figure 4).

**Figure 6:**
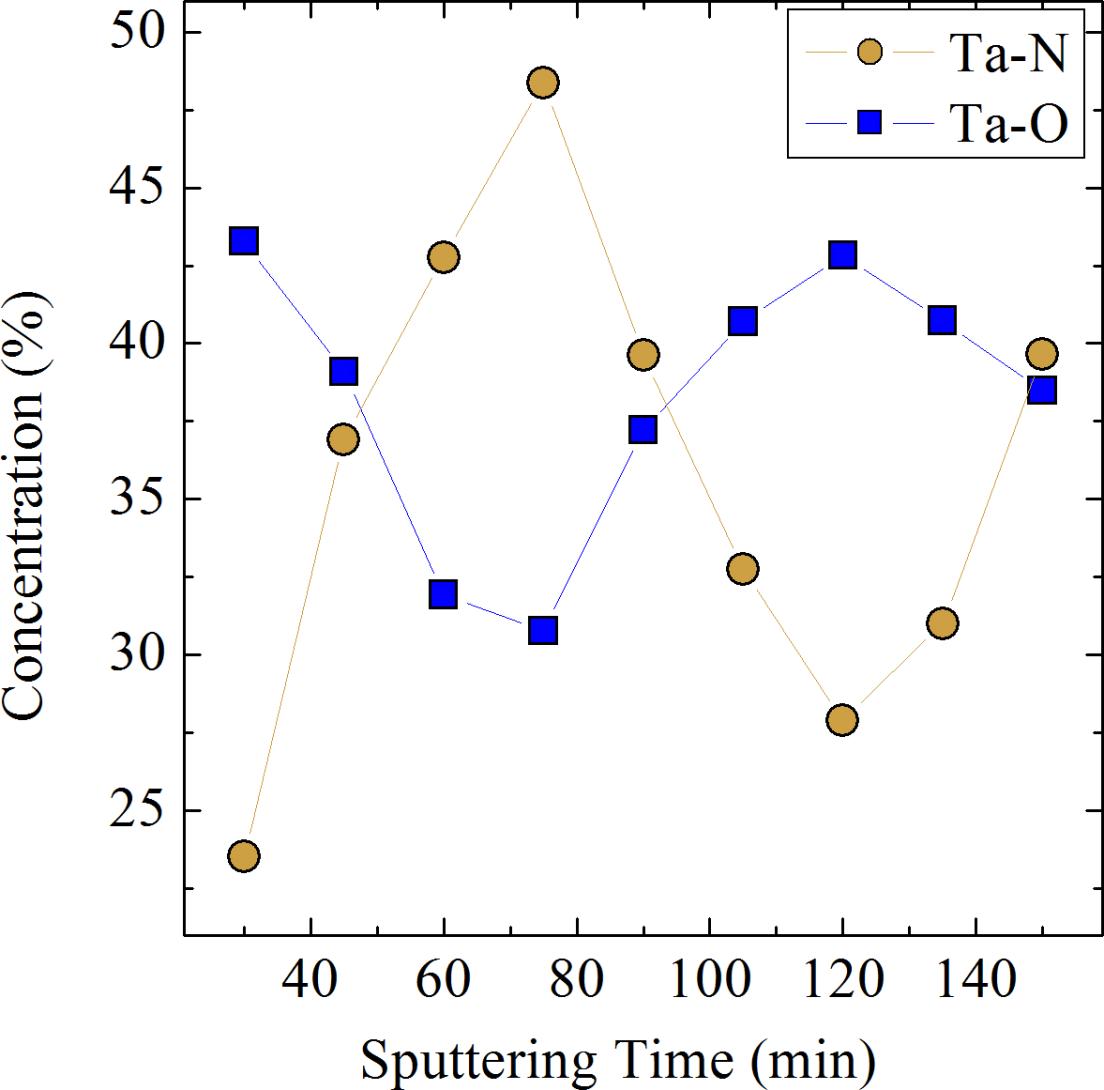
Depth distribution of chemical bonds in the ML12m9 sample obtained from XPS spectra acquired for different Ar^+^ sputtering times.

As we have shown in sections “GISAXS and XRR” and “GIXRD”, the ML12m9 sample contains a pure phase of Ta_2_N_3_ NCs, which are arranged in 2D planes, i.e., in the initially deposited metallic layers. To further examine the nature of the chemical bonding of Ta within this sample, we examine the in-depth XPS spectra for different Ar^+^ sputtering times. The results are shown in Figure 6, displaying the change of the fraction of Ta–N and Ta–O bonds with depth over one bilayer. Again, the periodic variation of the Ta–N concentration is consistent with the SIMS results from Figure 4, related to the formation of Ta_2_N_3_ NCs in the metallic layers. Concurrently, the relative concentration of Ta–O bonds shows a periodic behaviour as well, but with opposite phase, revealing the position of Ta atoms in the alumina spacer layers where they dominantly bond to oxygen. Therefore, we conclude that Ta_2_N_3_ NCs are immersed within an amorphous matrix composed of tantalum oxide and alumina. This is important for plasmonic applications of these MLs, as the resonant response of metallic NCs depends on the dielectric environment as well. On the other hand, designing and producing nanoplasmonic devices requires a full control over the phase composition of the dielectric media. In this context, our further efforts are directed toward minimizing the out-diffusion of tantalum atoms from the metallic layers.

## Conclusion

To summarize, we have grown at room temperature and subsequently annealed (Ta–N+Al_2_O_3_)/Al_2_O_3_ periodic multilayers, aiming at controlling the growth, size and spatial arrangement of tantalum nitride nanocrystals within an alumina matrix. We found tantalum-based nanoparticels already at 600 °C, with an average size increasing with increasing annealing temperatures. The nanoparticles remain amorphous up to 800 °C and crystallize in the bixbyite-Ta_2_N_3_ phase only at 900 °C. However, the arrangement and size of the Ta_2_N_3_ nanocrystals critically depend on the thickness of the metallic layers in MLs. We found a planar organization of Ta_2_N_3_ nanocrystals having 6 nm diameter for the metallic layers thicker than 12 nm. For thinner metallic layers, the nanocrystals became much bigger due to the mixing of metallic and alumina layers, while short-range ordering is absent.

It is known that the plasmonic properties of metal nanostructures are strongly dependent on the geometrical parameters, on the structure configuration and on the surrounding media of the nanostructures. By using the ML deposition procedure and annealing, we have demonstrated in this work a potential strategy to accomplish highly tunable and designable optical properties of thin films containing transition-metal nitride nanocrystals.

## Supporting Information

File 1 Additional experimental data.
Supporting Information File 1 shows representative 1D X-ray scattering profiles and corresponding fitting curves. Additional XPS spectra around the Ta 4p and N 1s core levels are also provided.
