# Supplementary material for: Ta2N3 nanocrystals grown in Al2O3 thin layers
Source: Beilstein J Nanotechnol. 2017 Oct 16;8:2162–70. doi: 10.3762/bjnano.8.215 (PMC5669238; doi:10.3762/bjnano.8.215)
Supplement: File 1 — Additional experimental data. Supporting Information File 1 shows representative 1D X-ray scattering profiles and corresponding fitting curves. Additional XPS spectra around the Ta 4p and N 1s core levels are also provided. [file Beilstein_J_Nanotechnol-08-2162-s001.pdf]

Supporting Information

for

Ta_2N_3 nanocrystals grown in Al_2O_3 thin layers

Krešimir Salamon*[1], Maja Buljan[1], Iva Šarić[2], Mladen Petravić[2] and Sigrid Bernstorff[3]

Address: [1]Rudjer Bošković Institute, Bijenička cesta 54, HR-10000 Zagreb, Croatia; [2]Department of Physics and Center for Micro- and Nanosciences and Technologies, University of Rijeka, R. Matejčić 2, 51000 Rijeka, Croatia and [3]Elettra-Sincrotrone Trieste, Strada Statale 14, km 163.5, I-34149 Basovizza (TS), Italy

Email: Krešimir Salamon* - ksalamon@irb.hr

*Corresponding author

Additional experimental data

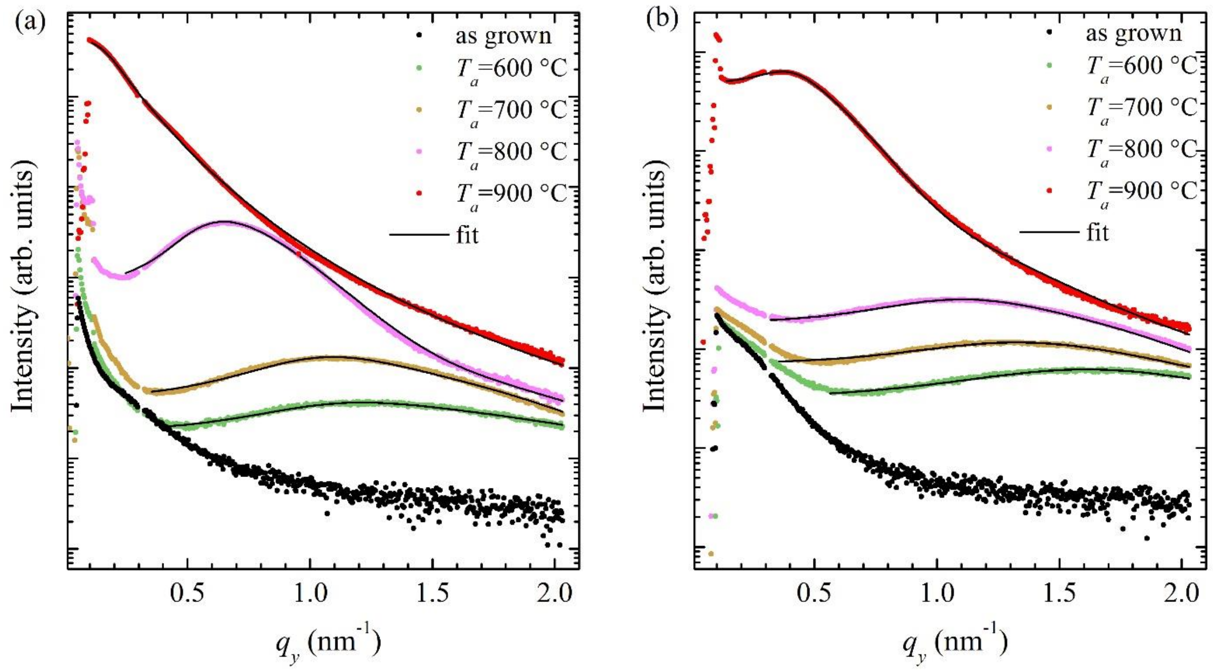

Figure S1: 1D intensity cross sections taken from the GISAXS patterns (shown in Figure 1) along the q_y axis (constant q_z=0.46 nm^{-1}) of as grown and annealed multilayers with (a) thinner (4 nm) and (b) thicker (12 nm) metallic layers. The spectra are vertically shifted for clarity. Solid lines represent best fits to the experimental data.

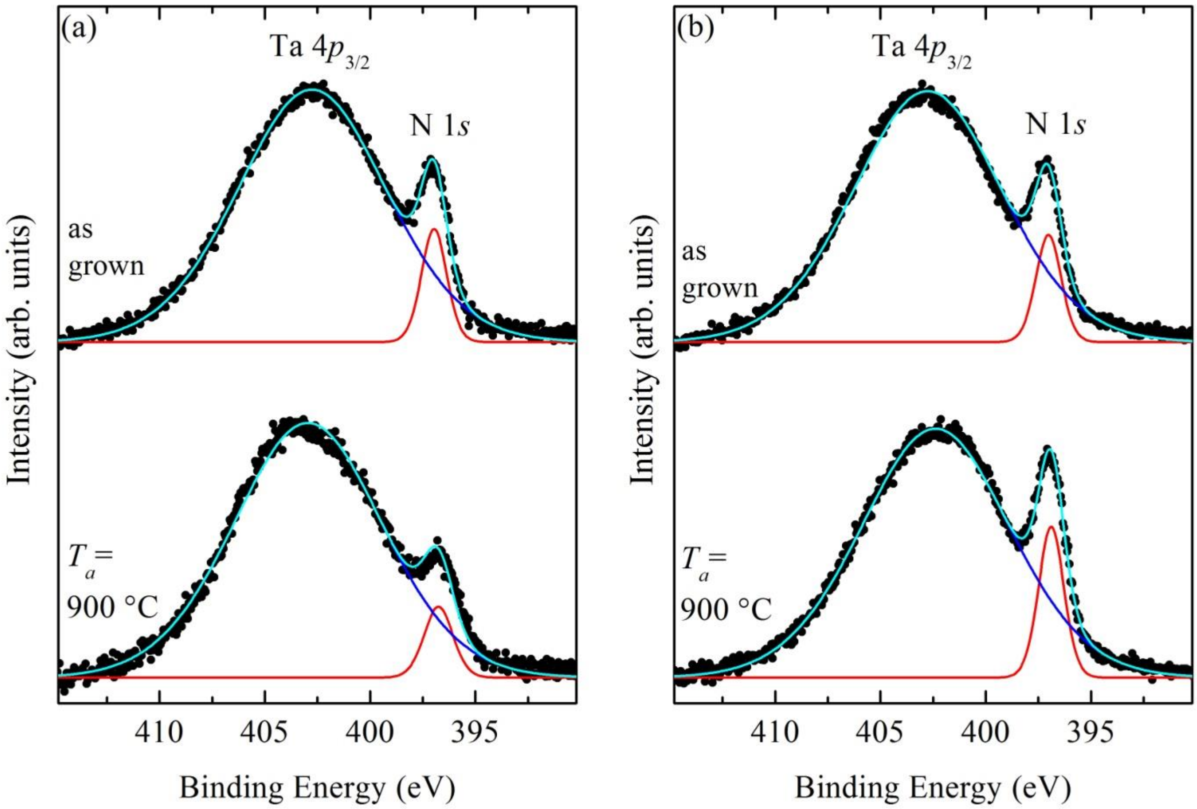

Figure S2: Deconvoluted XPS spectra for Ta 4*p* and N 1*s* core levels of the samples shown in Figure 5 of the main text: as grown (upper curves) and annealed at 900 °C (lower curves) MLs for two different nominal thicknesses of the metallic layers: (a) 4 nm (b) 12 nm. The location of N 1*s* energy level at BE of 397.2 eV implies the formation of N-Ta bonds [1].

References

[1] Lehn, J.-S. M.; van der Heide, P.; Wang, Y.; Suh, S.; Hoffman, D. M. *J. Mater. Chem.* **2004**, *14*, 3239–3245.
